# Somebody's Darling

**Published:** 1887-05-28

**Authors:** Marie Lacoste


### SOMEBODY'S DARLING.

Into a ward of white-washed halls,
Where the dead and the dying lay,
Wounded by bayonets, shells and balls,
Somebody's Darling was borne one day.
Somebody's Darling, so young and so brave,
Wearing yet on his pale sweet face,
Soon to be hid by the dust of the grave,
The lingering light of his boyhood's grace.

Matted and damp are the curls of gold,
Kissing the snow of that fair young brow;
Pale are the lips of delicate mould—
Somebody's Darling is dying now.
Back from his beautiful blue-veined brow
Brush all the wandering waves of gold;
Cross his hands on his bosom now—
Somebody's Darling is still and cold.

Kiss him once for Somebody's sake;
Murmur a prayer soft and low;
One bright curl from its fair mates take—
They were Somebody's pride, you know.
Somebody's hand had rested there;
Was it a mother's, soft and white?
And have the lips of a sister fair
Been baptised in the waves of light?

God knows best—He has Somebody's love;
Somebody's heart enshrined him there;
Somebody wafted his name above
Night and morn on the wings of prayer;
Somebody wept when he marched away,
Looking so handsome, brave, and grand;
Somebody's kiss on his forehead lay,
Somebody clung to his parting hand.

Somebody's waiting and watching for him,
Yearning to hold him again in her heart;
And there he lies, with his blue eye dim,
And the smiling, childish lips apart.
Tenderly bury the fair young dead,
Pausing to drop on his grave a tear;
Carve on the wooden slab at his head,
"*Somebody's Darling slumbers here.*"

MARIE LACOSTE.

